# Liquid Gallium Nanozyme Coatings Enable Sustained Nitric Oxide Generation With Antioxidant and Anti‐Inflammatory Functions

**DOI:** 10.1002/advs.77143

**Published:** 2026-08-13

**Authors:** Franco Centurion, Kang Lin, Shu Geng, Siti Nur Asyura Adzlan, Qingqing Fan, Federico Mazur, Ravindra Kokate, Priyank Kumar, Christina Cortez‐Jugo, Frank Caruso, Rona Chandrawati

**Affiliations:** ^1^ School of Chemical Engineering and Australian Centre for Nanomedicine (ACN) The University of New South Wales (UNSW Sydney) Sydney New South Wales Australia; ^2^ Department of Chemical Engineering The University of Melbourne Parkville Victoria Australia; ^3^ School of Chemical Engineering The University of New South Wales (UNSW Sydney) Sydney New South Wales Australia

**Keywords:** liquid gallium, metal‐phenolic coatings, nanozymes, nitric oxide

## Abstract

Artificial nanozymes, defined as nanomaterials that mimic enzyme‐like catalytic activity, emerge as adaptable tools for biomedical applications by uniting catalytic activity with structural stability and chemical versatility. Here, we introduce liquid gallium (Ga) as a catalytic center for nitric oxide (NO) generation and demonstrate its translation into a multifunctional coating. Ga nanoparticles were stabilized with tannic acid (TA) and embedded into a TA‐zirconium (TA‐Zr^4+^) metal‐phenolic network (MPN), producing robust, substrate‐independent films. Ga catalyzed the decomposition of *S*‐nitrosothiols (RSNOs) through electron transfer, enabling NO generation from both model donors such as *S*‐nitrosoglutathione (GSNO) and endogenous precursors in human umbilical vein endothelial cells (HUVECs), with activity retained over multiple cycles. The TA‐Zr^4+^ framework stabilized the coatings and contributed intrinsic antioxidant and anti‐inflammatory activities, resulting in a platform that amplified therapeutic outcomes. Functionally, the coatings displayed tunable NO generation, enhanced intracellular NO levels in HUVECs by ∼48%, reduced pro‐inflammatory cytokines TNF‐α and IL‐6 by ∼35% and ∼40%, respectively, under LPS stimulation, and supported endothelial biocompatibility. Together, these findings establish liquid Ga as an efficient catalyst for NO generation and present a design strategy that advances implant coatings from conventional NO donor‐based systems toward active, regenerative, and multifunctional therapeutic interfaces.

## Graphical abstract

Liquid gallium (Ga) acts as a catalytic center for nitric oxide (NO) generation within a tannic acid–zirconium metal–phenolic network coating. Stabilized Ga nanoparticles catalyze *S*‐nitrosothiol decomposition through surface electron transfer, enabling controlled NO production, enhancing endothelial biocompatibility, and providing antioxidant and anti‐inflammatory effects for multifunctional therapeutic implant coatings.

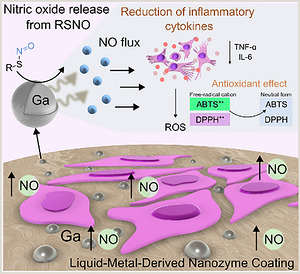

## 1 Introduction

Nitric oxide (NO) is a key mediator of vascular health, where it can regulate inflammation, modulate oxidative stress, and promote endothelial repair in a concentration‐ and context‐dependent manner [1, 2, 3, 4]. At controlled physiological levels, NO is associated with cytoprotective and anti‐inflammatory effects; however, excessive NO production or reactions with reactive oxygen species (ROS) can generate reactive nitrogen species, potentially contributing to oxidative and inflammatory damage. These context‐dependent effects highlight the importance of precise NO regulation. When maintained within a physiological range, NO generation can be beneficial for implantable devices operating in inflamed and oxidative microenvironments. However, achieving sustained and physiologically relevant NO delivery at implant surfaces remains a major challenge. Conventional NO‐releasing coatings, which rely on encapsulated NO donors such as diazeniumdiolates or *S*‐nitrosothiols (RSNOs) within polymeric or hybrid matrices, can achieve localized NO delivery but are hindered by burst release profiles, rapid donor depletion, and limited long‐term efficacy [5, 6, 7, 8].

Catalytic nanomaterials that mimic the activity of natural enzymes, known as nanozymes, offer a promising strategy to overcome the limitations of donor‐based NO delivery by generating NO in situ from endogenous precursors. Their intrinsic stability, tunability, scalability, and resistance to physiological degradation distinguish them from protein enzymes and have enabled advances in areas such as ROS regulation, antibacterial therapy, and cancer treatment [9, 10, 11, 12, 13, 14]. Several metal‐ and metal oxide‐based nanozymes, such as Cu [5], CeO_2_ [14], ZnO [15], Au [16], Pt [17], and Se [18] nanoparticles, have demonstrated the ability to catalyze NO generation from RSNOs, establishing a foundation for catalytic NO‐generating systems. More recently, emerging nanozyme systems have expanded beyond RSNO decomposition toward biomimetic catalytic pathways, including NOS‐like activity enabling NO generation from L‐arginine [19], as well as cascade catalytic platforms that achieve sustained and regulated NO production [20, 21]. However, most reported systems rely on rigid solid‐state surfaces and, in some cases, metal ion‐associated catalytic processes, and therefore still suffer from declining catalytic activity over time, susceptibility to biofouling, and concerns regarding metal ion leaching, which collectively limit their suitability for long‐term biomedical use [11]. For example, Cu‐based and metal‐organic framework (MOF)‐derived catalysts typically rely on redox‐active metal centers or structurally defined catalytic sites, where catalytic performance is closely dependent on the accessibility, coordination environment, and stability of these active sites. Maintaining long‐term catalytic activity and structural stability at biological interfaces remains an important consideration for such systems. Moreover, NO generation alone is often insufficient to address the complex inflammatory and oxidative microenvironment associated with vascular dysfunction and implant integration, highlighting the need for multifunctional nanozyme coatings that can generate NO catalytically and durably while simultaneously offering complementary antioxidant, anti‐inflammatory, and pro‐endothelialization functions.

Liquid metals (LMs), particularly low‐melting‐point gallium (Ga)‐based alloys, have recently emerged as a versatile platform for nanozyme development. Their unique combination of fluidic behavior, dynamic and self‐renewing interfacial chemistry, and resistance to fouling has enabled advances in industrial catalysis for reactions including CO_2_ reduction and hydrocarbon conversion [22, 23]. In biomedical contexts, the amphoteric oxide layer of Ga‐based LMs offers distinct catalytic properties, with the conductive metal core serving as an electron donor and the surface oxides acting as electron acceptors, together enabling redox transformations [24, 25]. Unlike conventional solid‐state nanozymes, this dynamic interface facilitates adaptive restructuring of catalytically active sites, potentially improving resistance to deactivation and enabling enhanced control over interfacial electron transfer. Despite these advantages, the exploration of LMs as nanozymes for catalytic NO generation from endogenous precursors remains largely unexplored. To our knowledge, no prior study has demonstrated the integration of LM‐derived NO nanozymes into implantable coatings for sustained and multifunctional therapeutic action.

Here, we demonstrate that liquid Ga exhibits robust enzyme‐like catalytic activity toward physiological RSNOs such as *S*‐nitrosoglutathione (GSNO), enabling controlled NO generation under physiological conditions. Systematic studies revealed concentration‐ and pH‐dependent activity with Michaelis–Menten kinetics, and density functional theory (DFT) simulations identified GaOOH as the most catalytically active surface species. Building on this catalytic function, Ga nanoparticles were stabilized with tannic acid (TA) and incorporated into a TA‐zirconium (TA‐Zr^4+^) metal‐phenolic network (MPN) to form robust, substrate‐independent coatings via spin coating. The MPN framework provided adhesion and stability while contributing intrinsic antioxidant and anti‐inflammatory properties. The resulting TA‐Zr^4+^‐Ga coatings exhibited tunable and sustained NO generation via catalytic decomposition of both exogenous GSNO and endogenous RSNOs in human umbilical vein endothelial cells (HUVECs). By integrating Ga catalytic reactivity with polyphenolic multifunctionality, this work advances implant coatings from conventional NO‐releasing films, which rely on finite NO reservoirs, toward an active, durable, and biologically multifunctional nanozyme system capable of controlling inflammation and mitigating oxidative stress.

## 2 Results and Discussion

### 2.1 Enzyme‐Mimetic Behavior of Liquid Ga Enables NO Generation From RSNOs

**FIGURE 1:**
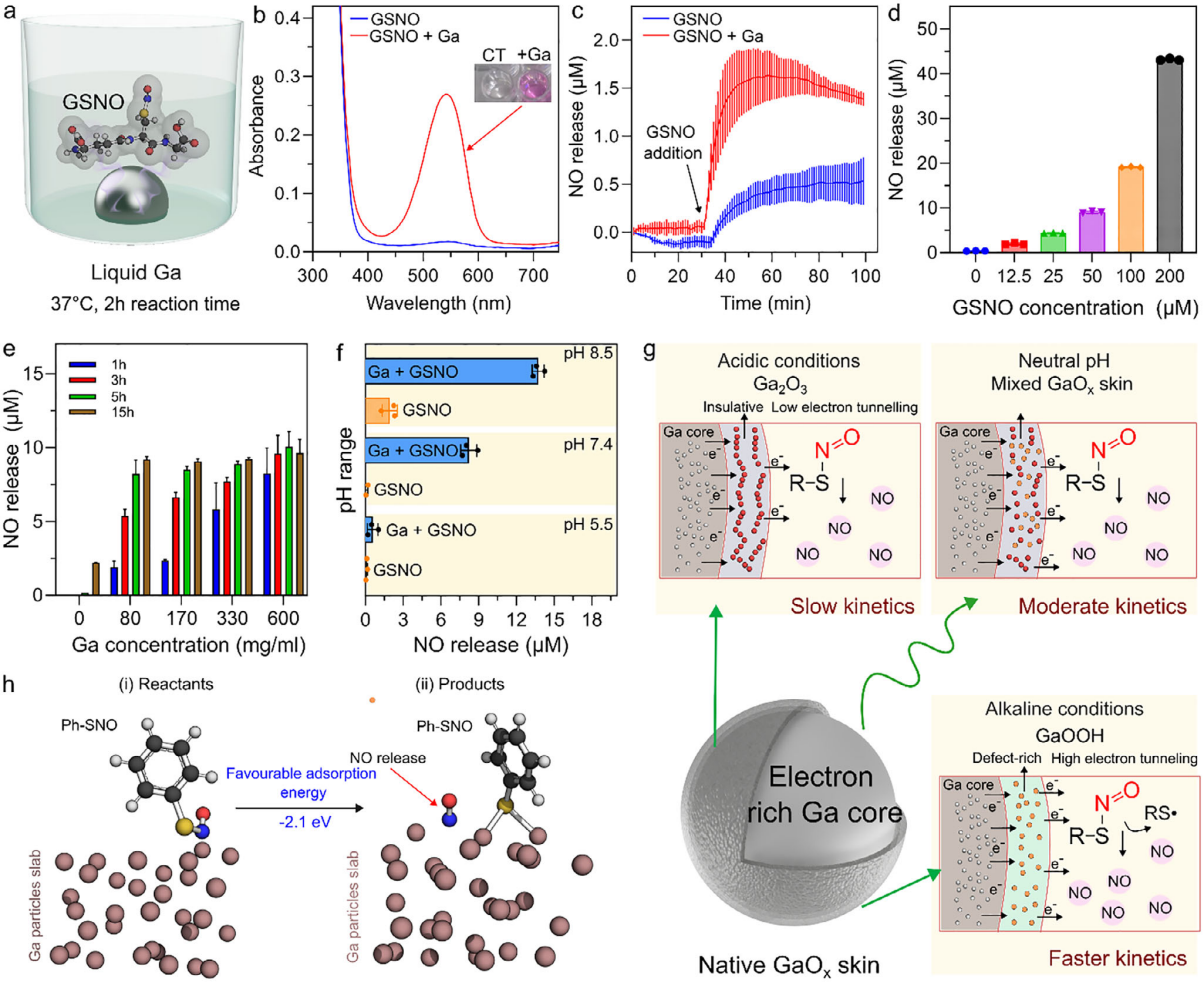
Overview of Ga‐mediated NO generation from GSNO, including catalytic performance and mechanistic insights. (a) Schematic illustration of the interaction between liquid Ga and GSNO. (b) Ga‐mediated NO generation from GSNO quantified using the Griess assay. (c) Real‐time electrochemical monitoring of Ga‐catalyzed NO generation from 50 µM GSNO in HEPES buffer (pH 7.4). (d) Concentration‐dependent NO generation from GSNO (0–200 µM). (e) Effect of Ga concentration on catalytic NO generation from 50 µM GSNO in HEPES buffer (pH 7.4) over 1–15 h. (f) Effect of pH (5.5, 7.4, and 8.5) on Ga‐catalyzed NO generation after 1 h of reaction. (g) Proposed mechanism underlying the pH‐dependent catalytic activity of Ga, highlighting the role of the GaO_x_ skin as a catalytic switch. (h) DFT‐calculated binding energy between Ph‐SNO and Ga. Data are presented as mean ± standard deviation, n = 3 independent experiments.

The catalytic interaction between liquid Ga and RSNOs was first investigated by introducing a Ga droplet into a GSNO solution under physiological conditions (Figure 1a). Ga was selected due to its low melting point (∼29.8°C) and biocompatibility, enabling its use in liquid form at physiological temperatures for in situ catalytic applications [26]. GSNO was chosen as it is a stable RSNO abundant in blood at micromolar concentrations [27], making it an appropriate model substrate for evaluating catalytic NO generation [28].

Upon addition of Ga to GSNO, a distinct color change was observed and confirmed by the Griess assay, indicating the formation of NO‐derived nitrite (Figure 1b). To further verify NO generation in real time, an NO‐sensitive electrochemical probe was used, which showed a clear increase in NO signal in the presence of Ga (Figure 1c). These findings demonstrate that liquid Ga can directly catalyze the decomposition of GSNO to generate NO under biologically relevant conditions.

To quantify this catalytic activity, NO generation was assessed across a range of GSNO concentrations (0–200 µm), revealing a clear concentration‐dependent response (Figure 1d). In a separate extended concentration‐range experiment (GSNO concentration up to ∼1700 µm), higher NO generation rates were observed, with a non‐linear behavior suggesting substrate affinity and catalytic turnover similar to enzyme‐like behavior (Figure S1). As shown in Figure S2a, the reaction velocity increased with substrate concentration and followed Michaelis–Menten kinetics, giving a V_max_ of 3.77 × 10^−14^ M s^−1^ mg^−1^ and a K_m_ of 584.1 µm with a good fit (R^2^ = 0.9548). The corresponding Lineweaver–Burk plot (Figure S2b) further confirms the linearity of the reciprocal transformation.

The catalytic activity of Ga was then systematically evaluated by varying both Ga concentration and pH to assess its enzyme‐mimetic specificity and adaptability. Increasing Ga concentration led to progressively higher NO generation from 50 µM GSNO (a concentration selected to enable reliable quantification of catalytic activity under controlled experimental conditions), with conversion efficiencies reaching ∼19% at 330–600 mg/mL after 1–15 h (Figure 1e). In contrast, negligible conversion was observed in the absence of Ga, confirming a dose‐dependent catalytic effect that plateaued at higher Ga levels. The catalytic performance was also strongly influenced by pH. The highest NO generation occurred under alkaline conditions (pH 8.5) (Figure 1f), where possibly the deprotonation of thiol/RSNO groups facilitates GSNO decomposition through more favorable surface redox reactions. At physiological pH (7.4), the system still retained substantial catalytic activity, highlighting the relevance of Ga for biologically compatible NO production. By contrast, acidic conditions (pH 5.5) markedly reduced the catalytic performance, consistent with protonation effects that limit RSNO dissociation and hinder electron transfer at the interface. This pH‐responsive catalytic profile could be attributed to Ga spontaneously forming a thin oxide/hydroxide shell around its electron‐rich metallic core in aqueous media [29]. The nature of this interfacial layer is highly pH‐dependent (Figure 1g): under alkaline conditions, it predominantly exists as GaOOH, a hydroxylated and defect‐rich phase that readily conducts electrons from Ga^0^ into the antibonding orbitals of RSNO, weakening the S─N bond and accelerating NO generation via RSNO decomposition. At acidic pH, however, the oxide layer is dominated by Ga_2_O_3_, which is denser and less conductive, restricting charge transfer and suppressing catalytic efficiency. These interfacial dynamics explain the observed pH‐responsive activity and reinforce the classification of Ga as an efficient catalyst for controlled NO generation.

To further elucidate the catalytic mechanism, DFT calculations were performed using a model nitrosothiol (Ph–SNO) and revealed a favorable adsorption energy on metallic Ga (−2.1 eV), supporting the proposed role of the metallic Ga core in facilitating RSNO decomposition and catalytic NO generation (Figure 1h). In contrast, Ph–SNO exhibited substantially stronger binding in the presence of GaOOH (−4.70 eV, Figure S3) and unfavorable adsorption energies from Ga_2_O_3_ (0.87 eV) (Figure S4). These results align with the experimental trends, demonstrating that the hydroxide‐rich GaOOH surface, which predominates under alkaline conditions, provides the most favorable environment for RSNO activation. In this phase, enhanced electron transfer from the Ga core through the defect‐rich hydroxide shell effectively weakens the S─N bond, facilitating NO generation via RSNO decomposition. Together, the experimental and computational results establish that the pH‐responsive surface chemistry of liquid Ga, particularly the formation of GaOOH, plays a central role in enabling efficient and controllable RSNO decomposition.

### 2.2 Translation of Ga Catalysis Into a Nanoparticulate Platform

**FIGURE 2:**
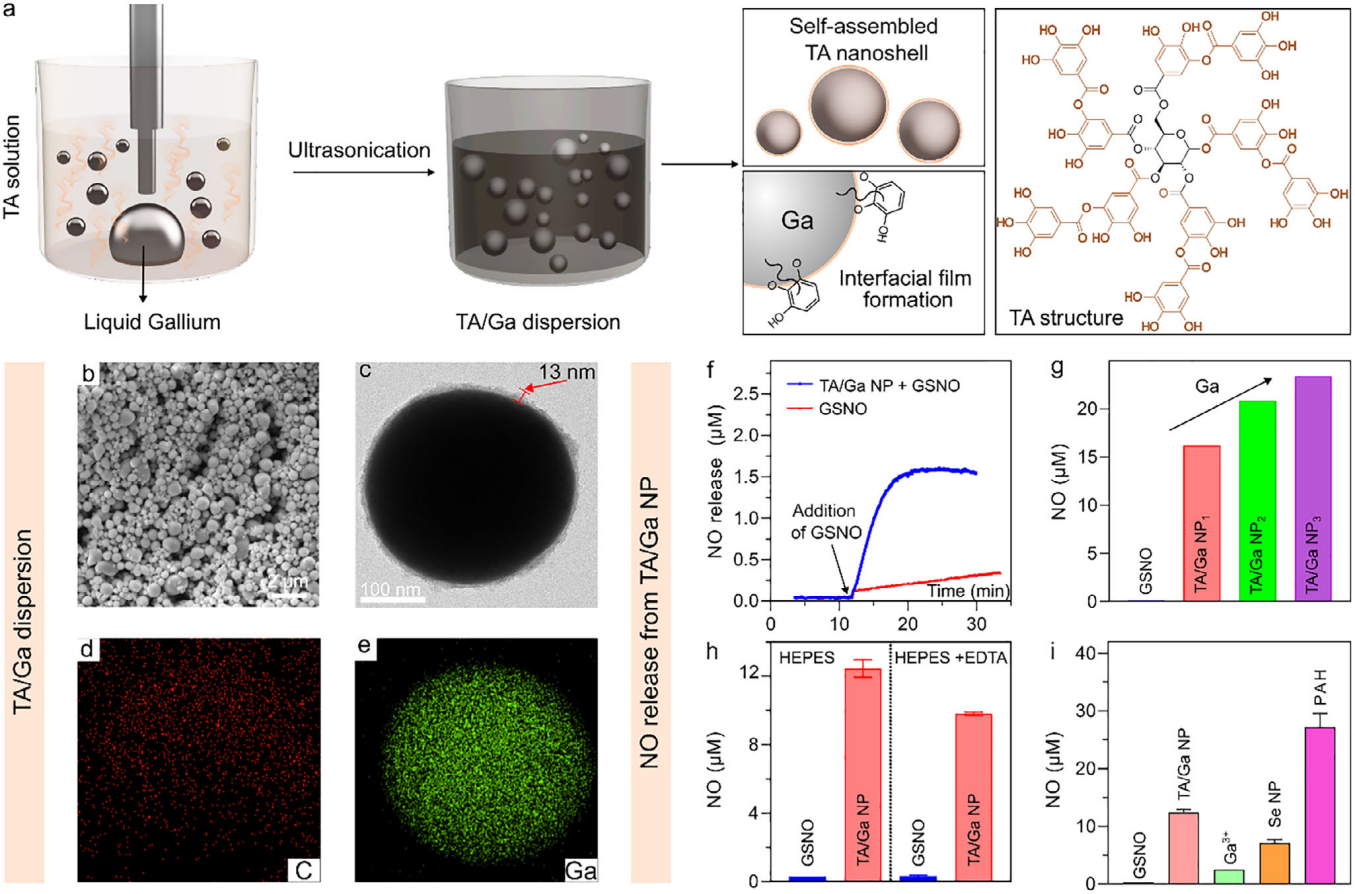
Synthesis, characterization, and catalytic NO generation from GSNO using TA/Ga nanoparticles (a) Schematic illustration of the synthesis of TA/Ga nanoparticles. (b) SEM image of the TA/Ga nanoparticles (scale bar = 2 µm). (c) High‐resolution TEM image showing a self‐assembled polyphenol (TA) coating on the TA/Ga nanoparticles (scale bar = 100 nm). (d,e) Elemental mapping (EDX) of C and Ga in the TA/Ga nanoparticles. (f) Real‐time electrochemical monitoring of NO generation from GSNO catalyzed by TA/Ga nanoparticles using a selective NO electrode. (g) Concentration‐dependent catalytic NO generation from GSNO by TA/Ga nanoparticles, quantified using the Griess assay (single measurements). (h) NO generation from GSNO by TA/Ga nanoparticles in HEPES buffer (pH 7.4) and HEPES/EDTA buffer (pH 7.4). (i) Comparison of NO generation by TA/Ga nanoparticles, Ga^3+^, selenium nanoparticles (SeNPs), and poly(allylamine hydrochloride) (PAH). Data are presented as mean ± standard deviation, n = 3 independent experiments unless otherwise stated.

Inspired by the dynamic catalytic reactivity of Ga toward GSNO, we sought to translate this activity into a more versatile nanoscale platform by engineering Ga‐based nanoparticles. To enable coating applications, Ga was first stabilized into nanoparticles using TA as a coordinating and stabilizing agent through probe sonication (Figure 2a). This approach was essential, as Ga alone is prone to rapid oxidation and aggregation in aqueous environments [30, 31], leading to the formation of unstable Ga oxide flakes. The resulting TA/Ga nanoparticles (Figure 2b and Figure S5) exhibited a uniform coating approximately 13 nm thick, as confirmed by transmission electron microscopy (TEM) analysis (Figure 2c–e and Figure S6). To determine whether Ga retained its catalytic ability following nanoparticle formation, we quantified NO generation from GSNO using an electrochemical NO probe (Figure 2f). TA/Ga nanoparticles showed a consistent NO generation with an initial NO flux of 3.9 × 10^−12^ mol s^−1^ mg^−1^ in the presence of 50 µm GSNO in 3 mL HEPES buffer (pH 7.4), calculated from a linear fit to the 12–16 min rise (slope ≈ 0.29 µm min^−1^). Consistently, Griess assay measurements confirmed NO generation across different TA/Ga nanoparticle concentrations (Figure 2g), indicating that the nanoscale TA coating did not inhibit the catalytic function. To assess whether free Ga^3+^ ions contributed to the observed NO generation, a control experiment using HEPES buffer containing EDTA was performed. Only minimal NO generation (∼3 µm) was detected from this condition (Figure 2h), suggesting that the catalytic activity was predominantly driven by the nanoparticle surface rather than ion leaching. We subsequently compared the catalytic efficiency of TA/Ga nanoparticles against selenium nanoparticles (SeNPs) [32] and amine‐rich polymers such as poly(allylamine hydrochloride) (PAH) [33] (Figure 2i). TA/Ga nanoparticles exhibited higher NO generation from GSNO than SeNPs, though they were less effective than PAH, positioning them as a moderate yet biocompatible catalytic platform. Overall, these results demonstrate that the catalytic activity of Ga was preserved after its transformation into TA–coated nanoparticles. This persistence reflects the intrinsic reactivity of Ga, as sonication produces nanoscale droplets with increased surface area that expose more Ga–oxide/hydroxide interfaces, while the underlying liquid metal core remains an electron‐rich reservoir. The TA coating may further stabilize the nanoparticles by chelating surface Ga^3+^ ions, a process that has been reported to slow or prevent full conversion into dense, insulating Ga_2_O_3_ [34]. As a result, the TA shell sustains a permeable, dynamic interface through which electrons from the Ga core can activate the S–N bond of RSNO, thereby maintaining efficient NO generation. This mechanistic rationale explains why the nanoscale TA/Ga system retains the NO‐generating functionality of bulk Ga while also achieving improved stability and multifunctionality.

### 2.3 Engineering a Substrate‐Independent Polyphenol‐Based TA–Zr^4+^–Ga Coating

**FIGURE 3:**
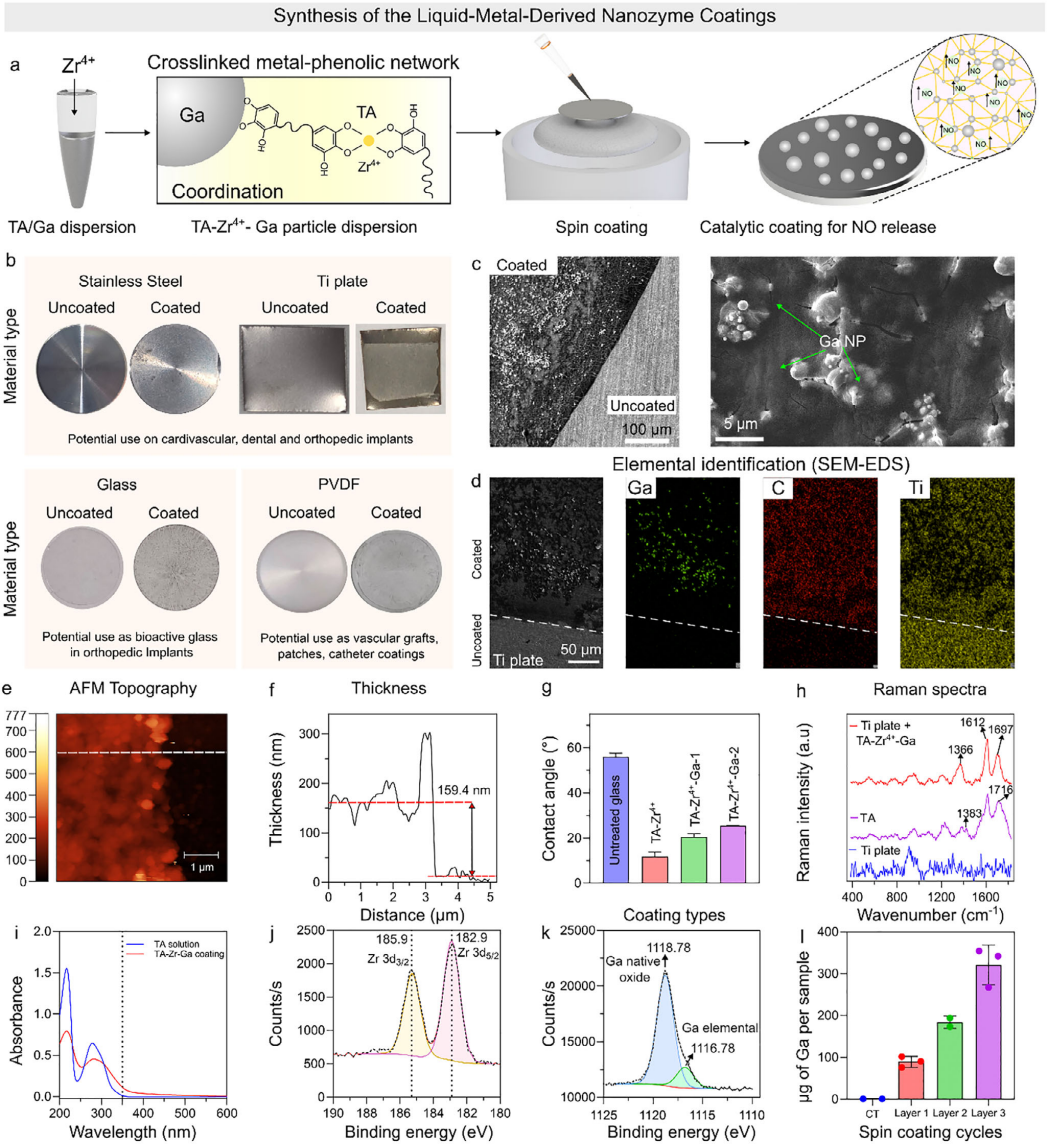
Fabrication, substrate‐independent adhesion, and physicochemical characterization of the TA‐Zr^4+^‐Ga liquid‐metal‐derived nanozyme coating. (a) Schematic illustration of the fabrication process of TA‐Zr^4^^+^‐Ga coatings via spin‐coating. (b) Demonstration of substrate‐independent adhesion of TA‐Zr^4^^+^‐Ga coatings on representative materials relevant to cardiovascular, dental, and orthopedic applications. (c) SEM images of the TA‐Zr^4^^+^‐Ga coating deposited on a Ti plate (scale bar = 100 and 5 µm) and (d) corresponding elemental mapping by SEM‐EDS (scale bar = 50 µm). (e) AFM topography of the TA‐Zr^4+^‐Ga coating on Ti plate (scale bar = 1 µm), with (f) thickness characterization. (g) Water contact angle characterization of the coatings. (h) Raman spectra of bare Ti, TA, and TA‐Zr^4+^‐Ga coatings. (i) UV–Vis absorption spectra of TA and TA‐Zr^4+^‐Ga coatings. (j) XPS analysis showing Zr 3*d* core‐level spectra and (k) Ga 2*p* and Zr 3*d* core‐level spectra. (l) Quantification of Ga loading per sample after one to three spin‐coating cycles via ICP‐OES. Data are presented as mean ± standard deviation, n = 3 independent experiments.

Upon confirming the NO‐generating capability of the TA/Ga nanoparticles, these nanomaterials were incorporated into a polyphenol‐based coordination matrix to construct a stable coating. This was achieved by exploiting the strong metal–phenolic coordination between TA and Zr^4+^, which forms a robust cross‐linked MPN [35, 36, 37, 38, 39] as evidenced by the formation of a soft gel (Figure S7). The TA‐Zr^4+^‐Ga nanoparticle dispersion was deposited onto substrates via spin‐coating (Figure 3a), with the sol remaining fluid long enough to enable uniform coverage before drying into a stable film. This strategy ensured that the catalytic activity of the TA/Ga nanoparticles was preserved while providing mechanical integrity. A key advantage of this coating system is its substrate‐independent adhesion. The TA‐Zr^4+^‐Ga coating adhered strongly and uniformly to a broad range of biomedically relevant materials, including stainless steel, titanium, glass, polyvinylidene fluoride (PVDF) membranes (Figure 3b), and also aluminum and PVC/DEHP surfaces (Figure S8). Furthermore, the coating was also successfully applied to non‐planar substrates such as Ti wires commonly used in stents (Figure S9). This versatility highlights its potential applicability across various implantable devices, such as cardiovascular stents, dental implants, and orthopedic prosthetics. The robust adhesive nature of the coating is primarily attributed to the chemical versatility of polyphenols, which are known to engage in a diverse array of interactions, including hydrogen bonding, hydrophobic effects, *π*–*π* stacking, and metal coordination, allowing them to bind effectively to both organic and inorganic surfaces [40].

To assess the structural integration and surface morphology of the coating, detailed characterization was performed using Ti substrates as a representative biomedical surface. Scanning electron microscopy (SEM) imaging of TA–Zr^4+^‐Ga‐coated Ti substrates confirmed the successful deposition of a conformal coating embedded with TA/Ga nanoparticles, forming a continuous and well‐integrated layer (Figure 3c). SEM‐EDS mapping of the coated Ti substrates showed clear signals from C and Ga, along with attenuation of the Ti signal from the bare substrate (Figure 3d), confirming effective surface coverage. Atomic force microscopy (AFM) analysis further indicated that the coating was uniform and conformal, with an average thickness of ∼159.4 nm (Figure 3e,f). The coating exhibited nanoscale surface roughness (∼30‐60 nm), which increased with Ga deposition and likely arises from nanoscale structuring associated with Ga incorporation during coating formation. Surface wettability was assessed using water contact angle measurements (Figure 3g and Figure S10). Bare glass has a moderate wettability, with contact angles of around 55°–60°. After coating with TA–Zr^4+^, the surface becomes highly hydrophilic, as evidenced by the drastic reduction of the contact angle to ∼10°. Introducing a Ga layer onto the Ta–Zr^4+^ coating (TA–Zr^4+^‐Ga‐1) slightly increases the contact angle (∼19°), while adding two Ga layers (TA–Zr^4+^‐Ga‐2) further raises it to ∼23°–27°. Raman spectroscopy of the TA–Zr^4+^‐Ga coating (Figure 3h) revealed key vibrational features indicative of metal‐polyphenol coordination. A prominent shift in the carbonyl (C═O) stretching vibration was observed, moving from 1716 cm^−1^ in free TA to 1697 cm^−1^ in the coating. This downshift is characteristic of metal ion coordination, in this case likely involving Zr^4+^, which interacts with the ester or carboxyl functional groups of TA, weakening the C═O bond and resulting in the spectral shift. Additionally, a peak at 1366 cm^−1^ was detected, corresponding to C─O stretching or ring‐breathing modes of the aromatic structure within TA [41]. The preservation of this peak alongside the C═O shift supports the formation of a metal–phenolic network while maintaining the structural integrity of the polyphenol backbone. Unlike the characteristic ligand‐to‐metal charge transfer (LMCT) band produced by TA–Ti^4+^ at 385 nm [37], TA–Zr^4+^ produced a weak band located at 350 nm (Figure 3i). To further confirm this metal coordination, the X‐ray photoelectron spectroscopy (XPS) spectrum confirmed the presence of C 1s, O 1s, Ga 2p, and Zr (3p and 3d) peaks (Figure S11), consistent with the expected coating components. In the high‐resolution Zr 3d spectrum (Figure 3j), characteristic doublet peaks were observed at binding energies of 182.9 eV (3d_5/2_) and 185.3 eV (3d_3/2_), with a spin–orbit separation of 2.4 eV. These features, relative to typical Zr 3d signals, indicate the formation of catechol‐ and/or gallol‐Zr^4+^ coordination bonds within the network, as previously described [38]. The Ga 2p spectrum with a peak at 1116.3 eV is assigned to elemental Ga^0^ in the particle core, while the peak at 1118.5 eV is attributed to Ga^3+^ species arising from the thin surface oxide layer (Figure 3k). Finally, the Ga content in the TA–Zr^4+^‐Ga coatings was quantified by inductively coupled plasma–optical emission spectroscopy (ICP‐OES), revealing a linear correlation with the number of spin‐coating cycles (Figure 3l). This demonstrates that varying the coating cycles provides a controllable strategy to tune Ga loading and, consequently, the NO‐generating catalytic activity.

### 2.4 Biocompatibility and Catalytic NO Generation From TA‐Zr^4+^‐Ga Coating

**FIGURE 4:**
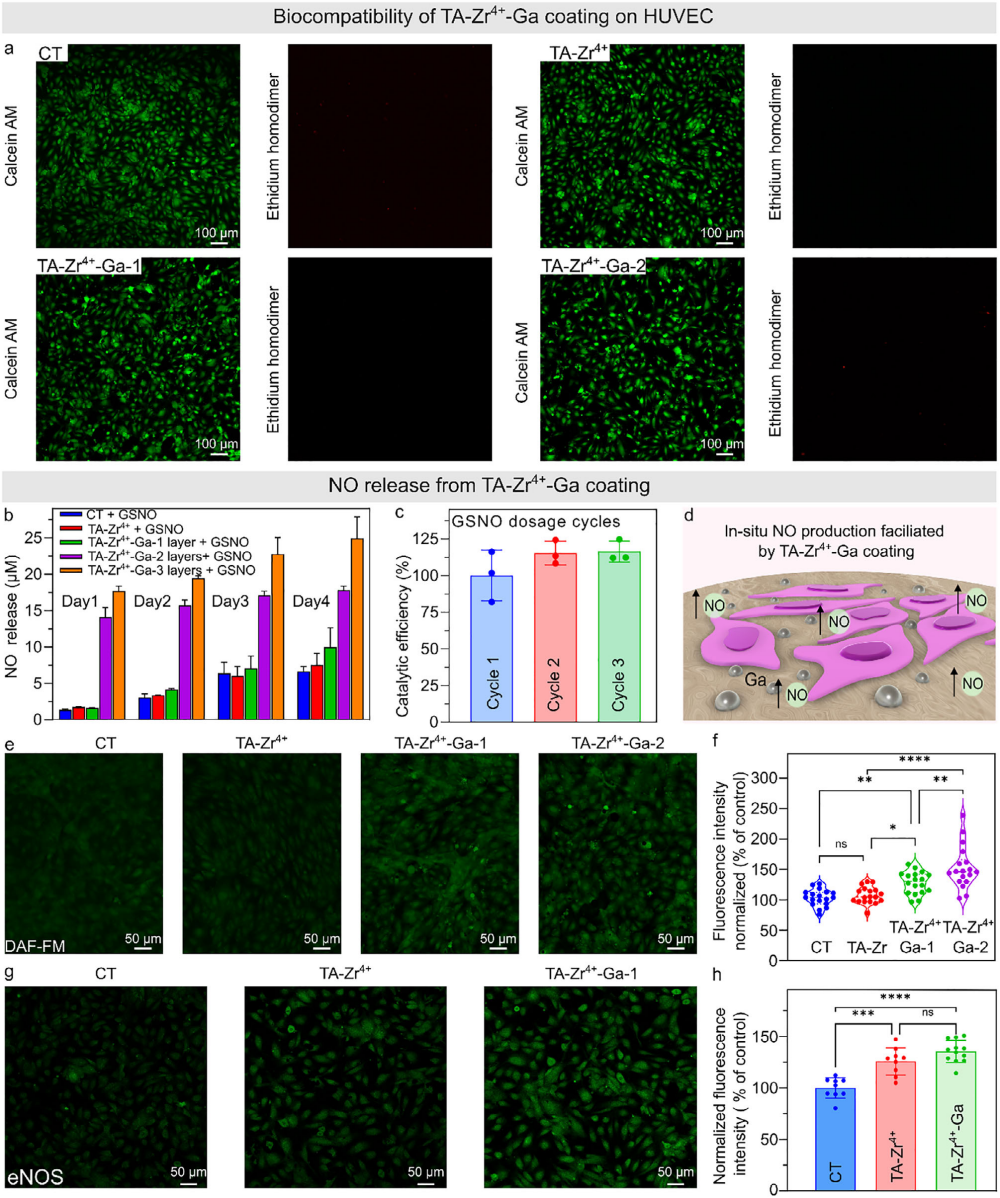
Biocompatibility, catalytic NO generation, and endothelial cell responses of TA–Zr^4+^–Ga coatings. (a) Live/Dead staining of HUVECs cultured on TA–Zr^4^^+^–Ga coatings, demonstrating good cytocompatibility (scale bar = 100 µm). (b) NO generation from TA–Zr^4+^–Ga coatings in the presence of exogenously added GSNO, showing the effect of Ga deposition layers. (c) Catalytic recyclability of the TA–Zr^4+^–Ga coatings over three consecutive NO‐generation cycles. (d) Schematic representation of the NO generation from the TA–Zr^4+^–Ga coatings in the presence of HUVECs. (e) Confocal microscopy images of HUVECs cultured with the TA–Zr^4+^–Ga coatings and stained with DAF‐FM (green) for intracellular NO detection (scale bar = 50 µm). (f) Corresponding DAF‐FM fluorescence intensity (% of control) analysis. (g) Confocal microscopy images of HUVECs cultured with the TA–Zr^4+^–Ga coatings and immunostained for eNOS expression (green) (scale bar = 50 µm). (h) Corresponding eNOS fluorescence intensity (% of control) analysis. Statistical significance was assessed using one‐way ANOVA with Tukey's post hoc test, ^*^ *p* <0.05, ^**^ *p* <0.01, ^***^ *p* <0.001, ^****^ *p* <0.0001. Data are presented as mean ± standard deviation, n = 3 biologically independent experiments, each performed in triplicate.

Biocompatibility was evaluated using live/dead staining of HUVEC and RAW 264.7 cells (Figure 4a and Figure S12) seeded on TA–Zr^4+^‐Ga–coated glass substrates (Figure S13), with the uniformity of Ga loading on each glass substrate confirmed across batches (Figure S14). Coatings with 1–2 deposition layers showed excellent compatibility with both cell lines, with only minimal cell death compared to bare glass controls. Assessment using Alamar Blue and CCK‐8 assays showed no significant reduction in cell viability compared with the control (Figures S15 and S16). In addition to cytocompatibility, the hemocompatibility of the coatings was evaluated to determine their suitability for blood‐contacting applications. After 2 h incubation with red blood cells, no detectable hemolysis or membrane disruption was observed, indicating excellent hemocompatibility (Figure S17). To investigate NO production, coatings with varying Ga content (1–3 deposition layers) were tested. As shown in Figure 4b, coatings with 2–3 Ga layers generated markedly higher NO output, producing a burst‐type release within the first 24 h and reaching a plateau around day 4. In contrast, coatings with a single Ga layer exhibited a more gradual and controlled NO output, indicative of a sustained release profile across the 4‐day period. As controls, coatings containing only TA–Zr^4+^ at different deposition layers (1‐3) did not show any catalytic effect for NO production (Figure S18), confirming that NO generation originated from Ga‐based catalysis. The catalytic activity of Ga was further validated under biologically relevant conditions. In serum‐containing cell medium, the coatings catalyzed significant NO production in the presence of GSNO (Figure S19). Next, the catalytic recyclability of the coated substrates was evaluated over three consecutive reaction cycles. The coatings retained their ability to catalyze NO generation with minimal loss of activity, thereby demonstrating both their efficiency and durability as a sustained NO‐generating platform (Figure 4c). To further assess catalytic durability, NO‐generating activity was evaluated over five consecutive cycles (Figure S20) and showed no abrupt decrease in signal, indicating that the response is not limited to a single‐use chemical reaction. XPS analysis after repeated GSNO exposure showed preservation of the characteristic Ga, Zr, O, and organic signals, supporting retention of the coating interface after repeated use (Figure S21). The structural stability of the coating was further evaluated under biologically relevant conditions. Time‐dependent ICP‐MS analysis in PBS supplemented with serum (up to 10 days) revealed low cumulative Ga release (∼13% of the total Ga loading), while Zr release remained negligible, consistent with the coordination stability of the TA‐Zr^4^^+^ network (Figure S22). Complementary SEM analysis before and after incubation (1 and 7 days) showed preserved morphology and surface coverage, with no evidence of significant degradation or delamination (Figure S23). In addition, coatings subjected to chemical (pH 3 and pH 9) and mechanical (sonication) challenges retained the characteristic TA–Zr coordination band and tannic acid absorption features in their UV–vis spectra, indicating that the coatings remained largely intact under these conditions (Figure S24). Together, these results support the structural stability of the coating and indicate limited metal ion exposure under biologically relevant conditions.

As a further mechanistic consideration, glutathione (GSH), a major intracellular thiol and reservoir for GSNO formation, can influence RSNO chemistry through transnitrosation reactions and thiol‐nitrosothiol equilibria. However, the catalytic activity of the TA‐Zr^4^^+^‐Ga coating does not rely on classical redox cycling of Ga^3^^+^, as Ga is considered redox‐inert under physiological conditions. Instead, NO generation is proposed to occur through surface‐mediated interactions between GSNO and gallium oxide/hydroxide species that facilitate RSNO decomposition. Therefore, while endogenous thiols such as GSH may modulate RSNO stability and NO availability in biological environments, they are not expected to be essential for the Ga‐mediated catalytic pathway. Future studies incorporating GSH and other biologically relevant thiols will provide further insight into the influence of complex thiol‐rich environments on NO generation and coating performance.

Following confirmation of material stability and NO‐generating capability, we investigated whether the coatings could promote NO generation at the cellular level. HUVECs were cultured on TA–Zr^4+–^Ga substrates without exogenous donors, thereby restricting NO sources to endogenous precursors (Figure 4d). After four days of culture, intracellular NO levels were assessed using the NO‐sensitive fluorescent probe DAF‐FM. Confocal imaging revealed a pronounced increase in intracellular fluorescence intensity for cells cultured on TA–Zr^4+^‐Ga coatings compared with uncoated controls and TA–Zr^4+^‐only surfaces (Figure 4e, Figures S25 and S26). Quantitative analysis showed a Ga‐content‐dependent enhancement in NO‐associated fluorescence, with TA–Zr^4+^‐Ga‐1 exhibiting an approximately 1.2‐fold increase (∼23.9%) and TA–Zr^4+^‐Ga‐2 an approximately 1.5‐fold increase (∼47.8%) in normalized fluorescence intensity relative to the control (Figure 4f). In contrast, no significant difference was observed between control and TA–Zr^4+^ surfaces, indicating that the increase in intracellular NO is specifically associated with the presence of Ga. These results demonstrate that TA–Zr^4+^‐Ga coatings can catalytically activate endogenous NO precursors in endothelial cells under biologically relevant conditions.

To probe a potential biological mechanism, a proof‐of‐concept immunofluorescence study was performed to assess endothelial nitric oxide synthase (eNOS), the enzyme responsible for endogenous NO production. Interestingly, the polyphenol‐only TA–Zr matrix induced upregulation of eNOS expression (Figure 4g,h), indicating a biologically mediated enhancement of endogenous NO biosynthesis, consistent with prior reports showing that polyphenol‐rich interfaces can promote a vasoprotective endothelial phenotype and enhance eNOS signaling through redox‐ and kinase‐associated pathways [42, 43]. In contrast, TA–Zr^4+^‐Ga coatings combined this biological effect with a pronounced increase in intracellular NO levels, consistent with an additional catalytic contribution. These observations suggest a dual mechanism for NO generation in TA–Zr^4+^‐Ga systems: (i) direct nanozyme‐mediated decomposition of endogenous RSNOs at Ga oxide/hydroxide interfaces, and (ii) stimulation of eNOS expression that enhances cellular NO production. Together, these complementary pathways may account for the observed NO signals and highlight the unique potential of TA–Zr^4+^‐Ga coatings to couple catalytic activity with endogenous enzymatic regulation, thereby amplifying therapeutic NO output.

### 2.5 Antioxidant and Anti‐Inflammatory Activity From TA–Zr^4+^‐Ga Coating

**FIGURE 5:**
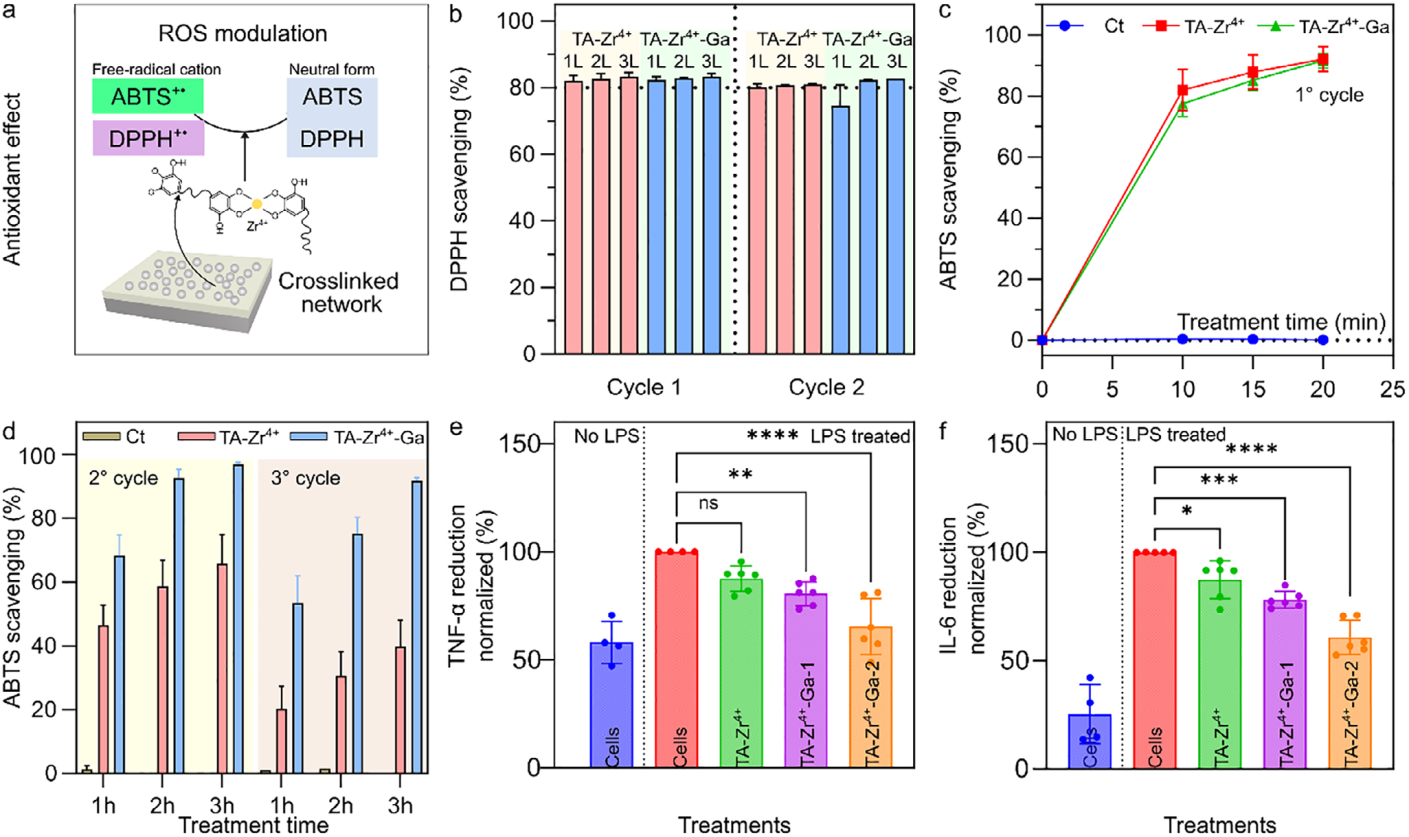
Antioxidant and anti‐inflammatory activities of TA–Zr^4^^+^–Ga coatings, demonstrating radical scavenging and modulation of inflammatory cytokine production. (a) Schematic illustration of the ROS scavenging ability of the TA–Zr^4+^–Ga coating against ABTS and DPPH radicals. (b) DPPH radical scavenging activity of the TA–Zr^4+^–Ga coatings across two consecutive cycles using different Ga deposition layers. (c,d) ABTS radical scavenging activity of the TA–Zr^4+^–Ga coatings over 1‐3 activity cycles. (e,f) Anti‐inflammatory activity of the TA–Zr^4+^–Ga coatings in LPS‐stimulated RAW 264.7 macrophages, showing modulation of TNF‐α and IL‐6 levels. Statistical significance was assessed using one‐way ANOVA with Tukey's post hoc test, ^*^ *p* <0.05, ^**^ *p* <0.01, ^***^ *p* <0.001, ^**^ ^*^ ^*^ *p* <0.0001. Data are presented as mean ± standard deviation, n = 3 independent experiments, each performed in duplicate.

Polyphenols are well known for their intrinsic antioxidant properties in nature, which have inspired their use in engineered biomaterials [44, 45, 46]. In this study, we evaluated the radical scavenging capacity of TA–Zr^4+^‐Ga nanocoating (Figure 5a), as oxidative stress is a critical factor contributing to vascular inflammation and restenosis in cardiovascular stents [47]. Using the DPPH assay, the coatings demonstrated efficient radical scavenging, achieving nearly 80% removal of DPPH radicals across two consecutive cycles (Figure 5b). Similarly, the ABTS assay revealed radical scavenging close to 90% in the first test cycle (Figure 5c). Interestingly, subsequent cycles highlighted a sustained and enhanced scavenging capacity specifically in coatings containing Ga, suggesting that TA primarily contributes to the initial antioxidant response, whereas Ga may provide a secondary mechanism that prolongs and amplifies radical neutralization (Figure 5d). This synergistic effect is significant, as conventional TA–Zr^4+^ coatings displayed strong initial activity but lacked the persistent radical scavenging observed in Ga‐containing coatings. The presence of Ga therefore likely reinforces the long‐term antioxidant barrier, a crucial property for cardiovascular stents where persistent oxidative stress could trigger smooth muscle proliferation, endothelial dysfunction, and in‐stent restenosis [47]. Supporting this, preliminary assessment of intracellular ROS levels in lipopolysaccharide (LPS)‐stimulated RAW 264.7 macrophages using DCFH‐DA staining (Figure S27) showed reduced fluorescence intensity following treatment with TA‐Zr^4^^+^ and TA‐Zr^4^^+^‐Ga coatings compared with LPS‐treated controls. While these observations suggest attenuation of oxidative stress at the cellular level, further quantitative validation is warranted. By enhancing antioxidant performance and NO generation features, TA–Zr^4+^‐Ga coatings may offer a therapeutic advantage over polyphenol‐only systems in preventing oxidative damage and maintaining vascular homeostasis. Since oxidative stress and inflammation are tightly interconnected, the enhanced antioxidant capacity of TA–Zr^4+^‐Ga coatings is expected to translate into anti‐inflammatory benefits. Inflammation is a major contributor to in‐stent restenosis, as pro‐inflammatory cytokines such as TNF‐α and IL‐6 drive endothelial dysfunction and smooth muscle proliferation [48]. One of the key protective roles of NO in the vasculature is its ability to suppress inflammatory signaling, inhibit macrophage activation, and reduce cytokine secretion [49]. Consistent with this, our coatings demonstrated strong anti‐inflammatory responses upon LPS stimulation on RAW 264.7 cells (Figure 5e,f). TA–Zr^4+^‐Ga coatings, which we previously showed to promote sustained NO production, significantly reduced TNF‐α and IL‐6 levels compared to TA–Zr^4+^ coatings. This effect was most pronounced with the TA–Zr^4+^‐Ga‐2 coating, which achieved ∼35% reduction in TNF‐α and ∼40% reduction in IL‐6 relative to the LPS‐stimulated control (100%). In contrast, TA–Zr^4+^ alone achieved only modest suppression around 12%. To further investigate cellular markers associated with the anti‐inflammatory effects of the coatings, we evaluated total cellular NF‐κB p65 fluorescence intensity and IL‐10 expression in LPS‐stimulated RAW 264.7 macrophages using immunostaining (Figures S28–S30). Total cellular NF‐κB p65 fluorescence intensity increased from 1.00 in control cells to 1.66 following LPS stimulation (∼66% increase). Treatment with TA‐Zr^4^^+^ coatings reduced the fluorescence intensity to 1.13–1.17 (∼30%–32% reduction relative to LPS stimulation), suggesting moderate suppression of pro‐inflammatory signaling. Incorporation of Ga resulted in a further loading‐dependent reduction in the total cellular NF‐κB p65 fluorescence signal, with levels decreasing to 0.98 at low Ga loading (∼13% additional reduction) and 0.85 at high Ga loading (∼27% additional reduction), approaching basal levels.

In parallel, IL‐10 expression exhibited a distinct response pattern. While LPS stimulation resulted in only a modest increase in IL‐10 expression (from ∼0.64 to ∼0.72), TA‐Zr^4+^ coatings alone did not significantly enhance IL‐10 levels. In contrast, TA‐Zr^4^^+^‐Ga coatings increased IL‐10 expression in a Ga‐loading‐dependent manner, with a modest increase at low loading (∼0.76) and a more pronounced elevation at high loading (∼1.00, corresponding to ∼35%–40% increase relative to LPS stimulation).

Together, these results provide further evidence of the anti‐inflammatory activity of the coatings. The TA‐Zr^4^^+^ coating appears to attenuate inflammation partly through reducing NF‐κB p65 fluorescence signal, consistent with its intrinsic antioxidant properties. Incorporation of Ga further decreased the total cellular NF‐κB p65 fluorescence signal while enhancing IL‐10 expression. This coordinated suppression of pro‐inflammatory signaling and promotion of anti‐inflammatory responses complements the observed reduction in TNF‐α and IL‐6 levels. These findings are consistent with a contribution of NO generation to the enhanced immunomodulatory activity of the Ga‐containing coatings.

## 3 Conclusion

This study identifies liquid Ga as a previously unrecognized enzyme‐mimetic catalyst capable of triggering NO generation from physiological *S*‐nitrosothiols and demonstrates its translation into stable TA‐Zr^4+^‐Ga coatings. By coupling Ga‐mediated catalytic NO generation with the intrinsic antioxidant and anti‐inflammatory functions of the MPN, these coatings provide tunable and sustained NO generation from both model donors and endogenous cellular precursors. Their multifunctional activity, biocompatibility, and hemocompatibility highlight strong translational potential for vascular grafts and other implantable devices. While the present study establishes the catalytic NO‐generating activity and biological performance of the TA‐Zr^4+^‐Ga coating under static conditions, further evaluation under physiologically relevant shear flow will be important to assess coating durability, catalytic performance, and biological function under dynamic blood‐contacting environments. In addition, comprehensive investigation of blood‐material interactions, including platelet adhesion, coagulation, protein adsorption, and long‐term performance under flow, will be required to further establish the suitability of this platform for blood‐contacting implant applications. Overall, this work introduces a strategy for integrating liquid metal nanozymes within MPN, providing a foundation for the development of durable, regenerative, and multifunctional therapeutic surfaces.

## 4 Experimental Section

### 4.1 Materials

Tannic acid (TA), zirconyl chloride octahydrate, Griess reagent (modified), phosphate‐buffered saline (PBS), Dulbecco's modified Eagle's medium (DMEM, high glucose), L‐glutamine (1%), Fetal Bovine Serum (FBS), HEPES buffer, ABTS, and 2,2‐diphenyl‐1‐picrylhydrazyl (DPPH) were purchased from Sigma–Aldrich. Hoechst 33342, DAF‐FM diacetate, eNOS polyclonal antibody, and LIVE/DEAD viability/cytotoxicity kit were obtained from Thermo Fisher Scientific Australia. Gallium (Ga, beads, 99%) was obtained from Roto Metals, USA. *S*‐nitrosoglutathione (GSNO) was purchased from MedChemExpress. Ultrapure water (18.2 MΩ cm) was provided by Arium pro Ultrapure Water Systems (Sartorius). All reagents were used without further purification.

### 4.2 Characterization

Characterization of the coatings involved a variety of analytical techniques. Raman spectroscopy was carried out using inVia Raman microscope (RENISHAW) equipped with a 532 nm laser source to analyze film composition. Surface topography and coating thickness were evaluated by atomic force microscopy (AFM) using a Bruker Dimension Icon operating in Scanasyst mode with a Scanasyst tip; raw data were processed using Nanoscope software. For elemental and morphological characterization, scanning electron microscopy (SEM) and energy‐dispersive X‐ray spectroscopy (EDS) mapping were conducted using a JSM‐IT500HR InTouchScope (JEOL) at accelerating voltages of 10 and 20 kV, respectively. The sample was characterized using transmission electron microscopy (JEOL F200 TEM) with an accelerating beam voltage of 200 kV. The compositions of samples were characterized by elemental mapping using TEM operating in high‐angle annular dark‐field scanning transmission electron microscope (HAADF‐STEM) mode. A water contact angle analysis was performed using an optical contact angle and surface tension meter (KSV CAM 200). X‐ray photoelectron spectroscopy (XPS) measurements were conducted using an ESCALAB250Xi spectrometer (Thermo Scientific, UK) with a monochromatic Al Kα X‐ray source (1486.68 eV, 120 W, 13.8 kV × 8.7 mA). Spectra were analyzed using Advantage software, and energy calibration was referenced to the C 1s peak at 284.8 eV. UV–Vis absorption spectra of the coatings were obtained using a Cary 5000 spectrophotometer (PerkinElmer) with the solid‐state accessory. The CLARIOstar Plus plate reader was used to quantify absorbance during ABTS and DPPH radical scavenging assays. Quantification of Ga elemental content of the coated coverslip with 1, 2, and 3 layers deposition was carried out via Inductively Coupled Plasma Optical Emission Spectrometry (ICP‐OES) using a PerkinElmer AVIO550. Spin‐coating was performed in a single step using a Laurell WS‐650Mz‐23NPPB system with the following parameters: 500 rpm acceleration, 6000 rpm speed, and 40 s spin time.

### 4.3 NO Generation From GSNO Triggered by Ga

Ga was first melted in a water bath maintained at 60°C. A 100 µL aliquot of molten Ga was carefully added to a 1 mL glass vial containing GSNO (50 µm) in HEPES buffer (10 mm, pH 7.4). HEPES buffer was selected instead of PBS to minimize phosphate‐induced RSNO decomposition and ensure stable and reproducible NO generation, as previously established for GSNO under physiologically relevant conditions [28]. A control sample without Ga was prepared under the same conditions. All samples were incubated at 37°C for 2 h on a horizontal shaker. Additional control samples containing Zr^4^^+^ and TA were also tested following the same protocol. NO output was quantified using the Griess assay, and results were corroborated using an electrochemical detection setup with a NO probe.

### 4.4 Quantification of NO Using the Griess Assay

The Griess assay was employed to quantify cumulative NO generation. A 100 µL aliquot of supernatant from the incubated samples was mixed with 100 µL of Griess reagent (40 mg mL^−^^1^ in HEPES buffer, 10 mm, pH 7.4) and incubated for 15 min in the dark. Absorbance was measured at 546 nm using a SpectraMax M5 microplate reader, and NO concentration was determined from a calibration curve.

### 4.5 Detection of NO Generation Using an Electrochemical Setup With a NO Probe

Real‐time monitoring of NO generation was performed using a free radical analyzer (TBR4100, WPI) with a NO‐sensitive probe (ISO‐NOP, WPI). Current changes were recorded and converted to NO concentrations using a calibration curve. The probe was immersed in 4 mL of HEPES buffer (10 mm, pH 7.4) with or without a Ga droplet (70 µL), followed by the addition of GSNO (7.4 µL, final concentration 50 µm). The reaction was carried out at 37°C, protected from light, and stirred at 400 RPM.

### 4.6 Evaluation of Ga‐Catalyzed NO Generation Under Variable Conditions

The NO‐generating properties of Ga (70 mg/mL) were initially evaluated using a concentration range of GSNO (0–200 µm) following 24 h incubation at 37°C under mild shaking (100 rpm) at pH 7.4 (10 mm HEPES buffer). To further characterize the system, kinetic studies were performed by varying the Ga concentration (0, 80, 170, 330, and 600 mg/mL) and monitoring NO production over different time points (1, 3, 5, and 15 h). Finally, the catalytic activity of Ga (600 mg/mL) was assessed under different pH conditions (5.5, 7.4, and 8.5) after 1 h of incubation at 37°C under mild shaking (100 rpm).

### 4.7 Computational Simulation of Ga and Ph‐SNO

All first‐principles calculations were performed within the framework of Density Functional Theory (DFT) using the Vienna Ab initio Simulation Package (VASP) [50, 51]. The theoretical foundation of DFT was based on the Kohn–Sham formalism [52]. Exchange–correlation effects were treated using the generalized gradient approximation (GGA) with the Perdew–Burke–Ernzerhof (PBE) functional [53]. The interaction between valence electrons and ionic cores was described using the projector augmented‐wave (PAW) method [54], and a plane‐wave basis set with a kinetic energy cutoff of 500 eV was applied throughout. Geometry optimizations were carried out until the residual forces on all unconstrained atoms were smaller than 0.15 eV/Å. Ionic relaxations were performed with the conjugate gradient algorithm (IBRION = 2), and electronic convergence was set to 1 × 10^−^^4^ eV. A Γ‐centered Monkhorst–Pack grid of 2 × 2 × 1 k‐points was used for Brillouin zone sampling, adjusted to the slab dimensions.

Adsorption energies (*E_ads_*) were calculated using the standard expression:

$$E_{ads}=E_{PhSNO+substrates}-\left[E_{PhSNO}+E_{substrates}\right]$$

where *E*_*PhSNO* + *substrates*_ is the total energy of the adsorbed system, *E_PhSNO_* is the energy of the isolated Ph‐SNO molecule in the gas phase, and *E_substrates_* is the energy of the clean Ga_2_O_3_ or GaOOH surface.

### 4.8 Preparation of TA–Ga Nanoparticles

A 50 µL (equivalent to 300 mg) aliquot of molten Ga was carefully added to a 50 mL Falcon tube containing a TA solution (10 mL, 5% w/v in ethanol). The mixture was then sonicated for 60 min using a probe sonicator (VCX 130, Sonics & Materials, Inc., 130 W, 20 kHz; tip model CV18) set at 60% amplitude with a pulsed mode (7 s on, 3 s off). Note: To prevent overheating, the tube was submerged in an ice‐water bath throughout the sonication process. In the next step, the resulting TA–Ga nanoparticles were aliquoted in 1 mL vials, and the excess of TA was removed by centrifugation (10 min at 10000 rpm). TA–Ga nanoparticle pellets were stored at 4°C before use.

### 4.9 NO Generating Capability Triggered by the TA–Ga Nanoparticles

To evaluate NO generation from TA–Ga nanoparticles, one TA–Ga nanoparticle pellet was resuspended in 500 µL of HEPES buffer (10 mm, pH 7.4) to obtain sample TA–Ga NP1. This suspension was then sequentially diluted 1:2 and 1:4 to prepare TA–Ga NP2 and TA–Ga NP3, respectively. Then, all the samples (100 µL) were diluted with HEPES (10 mm, pH 7.4) containing GSNO (50 µm) and incubated for 3 h. The Griess assay was used to quantify the NO output. To further study the possible contribution of Ga ions release into the system, the first experiment with TA–Ga‐NP1 was repeated using HEPES (10 mm, pH 7.4) supplemented with EDTA (0.1 mm) to evaluate the differences.

### 4.10 NO Generating Capability of the TA–Ga Nanoparticle in Comparison to Ga^3+^ Ions, SeNP, and PAH

TA–Ga nanoparticles (0.62 mg), SeNP (0.62 mg), Ga^3+^ (2.5 mg of Gallium nitrate, equivalent to 0.62 mg of Ga^3+^), and PAH polymer (0.62 mg) were redispersed in HEPES buffer (10 mm, pH 7.4) containing GSNO (50 µm, 1 mL) and incubated for 3 h. The Griess assay was used to quantify NO production.

### 4.11 Preparation of TA–Zr^4+–^Ga Coatings

A vial containing TA–Ga nanoparticles from the previous step was reconstituted with 600 µL of TA solution (2.5% w/v in a 1:1 water: ethanol mixture). Subsequently, 20 µL of zirconium oxychloride solution (0.59 m in DI water) was added, and the mixture was vortexed for 30 s, then left to rest at room temperature for 3 min. Finally, 10 µL of the resulting solution was spin‐coated onto the substrate at 6000 rpm with an acceleration of 500 rpm/s for a total of 40 s. The final coated substrate was dried at 90° for 1 h. Various substrates were selected for coating, including Ti sheets, aluminum, stainless steel, PVDF, PVC‐DEHP, glass, and titanium wires. For practical evaluation of the coating, glass substrates (13 mm, rounded) were primarily used.

### 4.12 Preparation of TA–Zr^4+^ Coating as Control

A TA solution (600 µL, 2.5% w/v in a 1:1 water: ethanol mixture) was mixed with zirconium oxychloride solution (20 µL, 0.59 m in DI water). The mixture was vortexed for 30 s and then allowed to rest at room temperature for 3 min. Subsequently, 10 µL of the solution was spin‐coated onto the substrate (i.e., glass coverslips) at 6000 rpm with an acceleration of 500 rpm/s for a total duration of 40 s.

### 4.13 Tuning the Ga Concentration on the TA–Zr^4+–^Ga Coatings

The Ga mass deposited on the substrates was increased by repeating the spin‐coating process, with samples labelled as 1 layer, 2 layers, and 3 layers to indicate the number of coating cycles. Three coating architectures (1, 2, and 3 deposition layers) were initially prepared to systematically investigate the effect of Ga loading on catalytic activity. Unless otherwise stated, subsequent biological studies focused on the 1‐ and 2‐layer coatings, which represent biologically relevant NO‐generating conditions identified from the mechanistic characterization.

### 4.14 Homogeneity of the Synthesis of the TA–Zr^4+–^Ga Coatings

TA–Zr^4+^‐Ga coatings were applied to 13 mm glass coverslips using a single deposition layer. Two independent batches were synthesized from scratch. Ga content was quantified by ICP‐OES. Briefly, each sample was digested with 3 mL of HNO_3_ at 40°C for 2 h, followed by 1 mL of H_2_O_2_ overnight. The next day, 1 mL of HCl was added, and the digestion continued for 2 h. Finally, samples were diluted with deionized water to a total volume of 10 mL prior to analysis.

### 4.15 Control of NO Production From the TA–Zr^4+–^Ga Coatings

TA–Zr^4+^‐Ga coatings were applied to 13 mm glass coverslips using 1, 2, or 3 deposition layers to achieve different Ga concentrations. The coated samples were then placed in 24‐well plates containing 50 µM GSNO in HEPES buffer (10 mM, pH 7.4) and incubated at 37 °C for designated time periods. The NO generation was evaluated using the Griess assay. In a similar manner, internal control containing TA–Zr^4+^ coatings with 1, 2, and 3 deposition steps were also tested for comparison.

### 4.16 Reusability of NO‐Generating Activity From TA–Zr^4+–^Ga Coatings

To assess the reusability of the TA–Zr^4+^‐Ga coatings to generate NO, a glass coverslip was coated with 3 deposition layers of TA–Zr^4+^‐Ga and incubated with GSNO (50 µm, 1 mL) for 2 h at 37°C under 100 rpm agitation. NO production was measured using the Griess assay. After each cycle, samples were washed with HEPES buffer, and reused for the next incubation with GSNO. In addition, the coating was also deposited on a silicon wafer under identical conditions, and XPS analysis was performed before and after five cycles of incubation with GSNO.

### 4.17 Study of Ga^3+^ and Zr^4+^ Release From TA–Zr^4+–^Ga Coatings

To evaluate potential ion leaching, TA–Zr^4+^‐Ga coatings (three Ga deposition cycles) were incubated in 5 mL of PBS supplemented with serum at 37°C for up to 10 days. At predetermined points (day 1, 3, 5, and 10), the supernatant was collected. The total concentrations of Ga and Zr in the supernatant were quantified by ICP‐MS.

To further assess whether released Ga^3+^ ions contribute to NO generation, a separate experiment was conducted. Coated samples with one and three Ga deposition cycles were incubated in 1 mL of HEPES buffer at 37°C. After 7 days, the supernatant containing any accumulated leached ions was collected and subsequently incubated with GSNO for 24 h. NO generation was quantified using the Griess assay.

### 4.18 Chemical and Mechanical Stability of the TA–Zr^4+–^Ga Coatings

The chemical and mechanical stability of the TA–Zr^4+^‐Ga coatings was systematically evaluated under physiologically relevant and accelerated conditions. For long‐term stability, coated samples were incubated in PBS supplemented with serum at 37°C for up to 10 days. Following incubation, the samples were gently rinsed with deionized water, dried, and the coating morphology was examined by SEM, with images acquired before and after incubation for comparison.

To assess chemical stability under extreme pH conditions, coatings (three Ga deposition cycles) were prepared on quartz substrates and exposed to buffered solutions at pH 3 and pH 9 for 1 h. After treatment, samples were rinsed thoroughly with deionized water and analyzed by UV–vis spectroscopy to confirm the presence and integrity of the coating based on its characteristic absorbance features.

Mechanical robustness was evaluated by subjecting coated samples to sonication in deionized water for 5 min. After sonication, samples were rinsed, dried, and characterized by UV–vis spectroscopy to assess any loss or degradation of the coating.

### 4.19 Biocompatibility of TA–Zr^4+–^Ga Coatings

The biocompatibility of TA–Zr^4^^+^–Ga coatings was evaluated using HUVEC and RAW 264.7 cells (below passage 20, ATCC). Cell viability was assessed via Live/Dead (Invitrogen) and AlamarBlue (Thermo Fisher) assays. RAW 264.7 macrophage cells were cultured in DMEM (Life Technologies) supplemented with 10% FBS, 0.25 mg/mL L‐Glutamine, and 1% Pen‐Strep (all Sigma–Aldrich), and sub‐cultured at 80%–90% confluency. Cells were seeded at 1 × 10^5^ cells/mL in 24‐well plates containing TA–Zr^4+^‐Ga‐coated glass coverslips and incubated at 37°C with 5% CO_2_ for 24 h. Note: All coated and uncoated coverslips were washed three times before use.

For Live/Dead assays, cells were stained with 2 µm calcein AM and 4 µm ethidium homodimer‐1, incubated for 30 min, washed with PBS, and imaged using a Zeiss LSM 800 confocal microscope. Live/dead cells were quantified with Imaris software, and viability was calculated as:

Cellviability(%)=Livecells/(Live+Deadcells)×100

For AlamarBlue assays, 100 µL of medium containing 10% AlamarBlue was added per well and incubated for 3 h. Fluorescence (Ex/Em = 560/590 nm) was measured with a SpectraMax M5, and viability was calculated as:

Cellviability(%)=Is/Ic×100

where Is = sample fluorescence; Ic = control fluorescence.

HUVEC cell viability was assessed using the same protocol.

### 4.20 Hemocompatibility of TA–Zr^4+–^Ga Coatings

Whole blood (collected in sodium heparin anticoagulant tubes) was obtained from three healthy adult donors (1 female and 2 males) following written informed consent, in accordance with approval from the Human Research Ethics Committee of the University of Melbourne (project no. 33501). The blood samples were then centrifuged at 3500 g for 5 min at room temperature to separate red blood cells (RBCs) from plasma. After centrifugation, the RBCs were washed three times with DPBS and resuspended in DPBS to obtain a 2% (v/v) RBC suspension. Uncoated or coated samples (on 13 mm glass coverslips) were immersed in 1.5 mL of 2% RBC suspension and incubated for 2 h at 37°C, with water as a positive control and DPBS as a negative control. High‐density polyethylene (HDPE) was additionally included as a negative reference material in accordance with ISO 10993–4 recommendations. After incubation, all samples were centrifuged at 9000 g for 5 min, and 100 µL of the sample supernatant was collected and transferred to a 96‐well plate. The absorbance of the supernatant was measured using a microplate reader at 577 nm. The hemolysis percentage of the tested materials was calculated using the following equation:

$$Hemolysis\;\left(\%\right)=\frac{A_{1}-A_{0}\;}{A_{w}-A_{0}}\times 100\%$$

where A_0_ is the absorbance of blood in DPBS, A_w_ is the absorbance of blood in water, and A_1_ is the absorbance of treated blood, all measured at 577 nm.

### 4.21 Intracellular NO Generation in HUVEC Cells Triggered by TA–Zr^4+–^Ga Coatings

HUVECs were seeded in 24‐well plates at a density of 5 × 10^4^ cells/mL onto TA–Zr^4+^‐Ga‐coated samples. Cells were cultured in complete HUVEC growth medium consisting of Endothelial Cell Growth Medium‐2 (EGM‐2, CC‐3156, Lonza) supplemented with EGM‐2 BulletKit components (EGM‐2 MV Microvascular Endothelial Cell Growth Medium‐2 SingleQuots Kit, CC‐4147, Lonza). After 4 days of incubation at 37°C in a humidified atmosphere with 5% CO_2_, intracellular NO generation was assessed using the molecular probe DAF‐FM diacetate (5 µm). In parallel, cell nuclei were stained with Hoechst 33342 at a 1:500 dilution. Fluorescence images were acquired and processed using Imaris software (Oxford Instruments) to quantify NO‐related fluorescence intensity and visualize nuclear morphology.

### 4.22 Endothelial Nitric Oxide Synthase (eNOS) Expression on HUVEC Cells Triggered by TA–Zr^4+–^Ga Coatings

To assess eNOS expression in HUVECs exposed to the coatings, HUVECs were seeded in 24‐well plates at a density of 5 × 10^4^ cells/mL onto TA–Zr^4+^‐Ga‐coated glass samples. Cells were cultured in complete HUVEC growth medium consisting of Endothelial Cell Growth Medium‐2 (EGM‐2, CC‐3156, Lonza) supplemented with EGM‐2 BulletKit components (EGM‐2 MV Microvascular Endothelial Cell Growth Medium‐2 SingleQuots Kit, CC‐4147, Lonza). After 4 days of incubation at 37°C in a humidified incubator with 5% CO_2_, cells were fixed with 2% paraformaldehyde (PFA) in PBS for 2 h at room temperature and stored overnight at 4°C.

The next day, fixed cells were permeabilized using 0.2% Triton X‐100 in PBS for 30 min at room temperature. Samples were then blocked with 2.5% bovine serum albumin (BSA) in PBS (300 µL per well) for 30 min. After blocking, cells were incubated overnight at room temperature with the primary anti‐eNOS antibody, prepared by diluting 1 µL of antibody in 300 µL of 1% BSA (40 µL applied per sample). Fluorescence images were acquired and processed using Imaris software (Oxford Instruments).

### 4.23 Antioxidant Activity of TA–Zr^4+–^Ga Coatings

#### 4.23.1 DPPH Radical‐Scavenging Activity Assay

The radical scavenging activity of TA–Zr^4^^+^–Ga coatings was assessed using the DPPH free radical method with slight modifications from a previous report [55]. Glass coverslips (13 mm) coated with 1, 2, or 3 layers were used. Each coated sample was treated with 500 µL of 0.2 mm DPPH in 95% ethanol and incubated in the dark at room temperature for 30 min. After incubation, aliquots were transferred to a 96‐well plate, and absorbance was measured at 531 nm. The scavenging activity (%) was calculated using:

$$\begin{array}{ccc} \mathrm{Radical}\;\mathrm{scavenging}\;\mathrm{activity}\;\left(\%\right) & = & \left(\left(A_{\mathrm{control}}-A_{\mathrm{sample}}\right)\;/\;A_{\mathrm{control}}\right)\\ & & \times 100 \end{array}$$

where A_control_ is the absorbance of the DPPH solution without the sample, and A_sample_ is the absorbance after treatment with the coated substrate.

#### 4.23.2 ABTS Radical‐Scavenging Activity Assay

ABTS solution (7 mm) was activated by incubating with 2.45 mm potassium persulfate overnight to generate ABTS radicals. TA–Zr^4+^‐Ga coatings and Ga‐free controls were then tested. The absorbance at 734 nm was recorded over 20 min and during additional cycles up to 3 h. Radical scavenging activity (%) was calculated using the following equation:

$$\begin{array}{ccc} \mathrm{Radical}\;\mathrm{scavenging}\;\left(\%\right) & = & \left(\left(\mathrm{Ab}s_{\mathrm{control}}-\;\mathrm{Ab}s_{\mathrm{sample}}\right)/\mathrm{Ab}s_{\mathrm{control}}\right)\\ & & \times 100 \end{array}$$

### 4.24 Detection of Intracellular ROS Production

RAW264.7 mouse macrophages (passage 11) were seeded at a density of 1 × 10^5^ cells per well onto bare glass coverslip, TA–Zr^4+^, and TA–Zr^4+^‐Ga‐coated glass coverslips in 24‐well plates and cultured in complete medium at 37°C under 5% CO_2_ for 18 h. Following cell attachment, the medium was replaced, and cells were stimulated with lipopolysaccharide (LPS, 1 µg/mL) for 24 h. The supernatant was removed, and cells were then cultured in a serum‐free DMEM medium containing 10 µm DCFH‐DA at 37°C for 45 min and washed three times with PBS. Finally, DCFH‐DA fluorescence was observed under a fluorescence microscope.

### 4.25 Anti‐Inflammatory Response of TA–Zr^4+–^Ga Coatings

RAW264.7 mouse macrophage cells (1 × 10^5^ cells/well, passage 11) were seeded onto TA–Zr^+4^, and TA–Zr^4+^‐Ga‐coated glass coverslips in 24‐well plates and cultured in complete medium at 37°C with 5% CO_2_ for 18 h. After incubation, the medium was removed, and cells were stimulated with lipopolysaccharide (LPS, 1 µg/mL) for 24 h. The levels of IL‐6 and TNF‐α released into the supernatant were quantified using ELISA kits (Abcam, ab222503 for IL‐6 and ab108910 for TNF‐α), following the manufacturer's protocol. Samples were diluted 1:2 for TNF‐α and 1:5 for IL‐6. Note: No addition of an exogenous NO donor was added.

### 4.26 Immunofluorescence Staining and Imaging of IL‐10 and NF‐κB p65 Signal

RAW264.7 mouse macrophages (1 × 10^5^ cells/well, passage 11) were seeded onto bare glass, TA–Zr^4^^+^‐, or TA–Zr^4^^+^–Ga‐coated glass coverslips in 24‐well plates and cultured in complete medium at 37°C under 5% CO_2_ for 18 h. After incubation, the medium was removed, and cells were then stimulated with lipopolysaccharide (LPS, 1 µg/mL) for 24 h, while cells cultured on bare glass without LPS served as the untreated control. Following treatment, the cells were fixed with 4% paraformaldehyde for 2 h, permeabilized with 0.2% Triton X‐100 for 30 min, and blocked with 2.5% bovine serum albumin for 30 min prior to immunostaining. Cells were then incubated with primary antibodies for interleukin‐10 (Alexa 647 conjugated IL‐10) (501412, BioLegend, 1:100), nuclear factor kappa B (NF‐κB) p65 (51‐0500, Thermo Fisher, 1:300) for 2 h, followed by incubation with secondary antibodies donkey anti‐rabbit Alexa Fluor 488 (A21206, Thermo Fisher, 1:400), and nuclear stain Hoechst 33342 (1:1000) for 2 h. Cells were rinsed three times with DPBS between each step, and imaging was performed on Zeiss LSM 800 confocal microscopes. All fluorescence imaging data were processed using ImageJ software. Mean fluorescence intensity was measured on a per‐cell basis.

### 4.27 Statistical Analysis

Quantitative data are presented as mean ± standard deviation (SD), unless otherwise stated. The sample size (n) is specified in each figure caption and generally represents n = 3 independent replicates per group. Where indicated, fluorescence intensities were normalized to the untreated control and expressed as a percentage or fold change relative to the control. For comparisons involving more than two groups in Figures 4 and 5 and the corresponding Supporting Information figures, statistical significance was assessed using ordinary one‐way analysis of variance (ANOVA), followed by Tukey's multiple‐comparisons post hoc test. Tukey‐adjusted *p* values were used, with statistical significance defined as α = 0.05. Statistical significance is denoted as ^*^ *p* <0.05, ^**^ *p* <0.01, ^***^ *p* <0.001, and ^****^ *p* <0.0001. Statistical analyses were performed using GraphPad Prism 11 (GraphPad Software, Boston, MA, USA).

## Author Contributions

**Franco Centurion**: conceptualization, methodology, investigation, writing – original draft, writing – review and editing. **Kang Lin**: methodology, investigation. **Shu Geng**: investigation. **Siti Nur Asyura Adzlan**: investigation. **Qingqing Fan**: methodology, investigation. **Federico Mazur**: investigation. **Ravindra Kokate**: methodology, investigation. **Priyank Kumar**: methodology, supervision. **Christina Cortez‐Jugo**: methodology, supervision. **Frank Caruso**: methodology, supervision. **Rona Chandrawati**: conceptualization, methodology, supervision, resources, funding acquisition, writing – review and editing.

## Conflicts of Interest

The authors declare no conflicts of interest.

## Supporting information




**Supporting File**: advs77143‐sup‐0001‐SuppMat.docx.

## Data Availability

The data that support the findings of this study are available from the corresponding author upon reasonable request.
