# Supplementary material for: Liquid Gallium Nanozyme Coatings Enable Sustained Nitric Oxide Generation With Antioxidant and Anti‐Inflammatory Functions
Source: Adv Sci (Weinh). 2026 Aug 13:e77143. Online ahead of print. doi: 10.1002/advs.77143 (PMC13470296; doi:10.1002/advs.77143)
Supplement: Supplementary file 1 [file ADVS-9999-e77143-s001.docx]

Supporting Information

**Liquid Gallium Nanozyme Coatings Enable Sustained Nitric Oxide Generation with Antioxidant and Anti-Inflammatory Functions**

Franco Centurion, Kang Lin, Shu Geng, Siti Nur Asyura Adzlan, Qingqing Fan, Federico Mazur, Ravindra Kokate, Priyank Kumar, Christina Cortez-Jugo, Frank Caruso, and Rona Chandrawati*

Franco Centurion, Kang Lin, Shu Geng, Siti Nur Asyura Adzlan, Federico Mazur, and Rona Chandrawati

School of Chemical Engineering and Australian Centre for Nanomedicine (ACN), The University of New South Wales (UNSW Sydney), Sydney, NSW 2052, Australia

E-mail: rona.chandrawati@unsw.edu.au

Franco Centurion

Present address:

Institute for Biomedical Materials and Devices (IBMD), Faculty of Science and ARC Centre of Excellence in Quantum Biotechnology (QUBIC), Institute for Biomedical Materials and Devices (IBMD), Faculty of Science, University of Technology Sydney, Sydney, NSW 2007, Australia

Qingqing Fan, Christina Cortez-Jugo, Frank Caruso

Department of Chemical Engineering, The University of Melbourne, Parkville, VIC, 3010 Australia

Ravindra Kokate, Priyank Kumar
School of Chemical Engineering, The University of New South Wales (UNSW Sydney), Sydney, NSW 2052, Australia

The Supporting Information is organized into the following sections to support the main findings of this study:

1. Catalytic NO generation and mechanistic studies (Figures S1–S4)
2. Material characterization (Figures S5–S14)
3. Biocompatibility and hemocompatibility (Figures S12, S15–S17)
4. NO generation performance and durability (Figures S18–S21)
5. Coating stability and metal leaching (Figures S22–S24)
6. Cellular responses (Figures S25–S30)

**Figure S1.** NO generation increases with GSNO concentration, indicating substrate-dependent catalytic activity of Ga. NO generation catalyzed by liquid Ga across a range of GSNO concentrations (0 -1800 µM) at 37°C. Data are presented as mean ± standard deviation from three independent experiments (n = 3).

Vmax: 3.77 x 10^-14^.
Km: 584.1

R^2^: 0.9548

R^2^: 0.9548

a

b

**Figure S2.** Ga-catalyzed NO generation follows Michaelis–Menten kinetics, consistent with enzyme-like behavior. (a) Michaelis–Menten analysis of Ga-catalyzed NO generation from GSNO at 37 °C. (b) Corresponding Lineweaver–Burk plot derived from the Michaelis–Menten kinetic data. Data are presented as mean ± standard deviation from three independent experiments (n = 3).

**Figure S3.** DFT results suggest NO dissociation occurs upon RSNO adsorption on GaOOH surfaces. Optimized structure of Ph-SNO adsorbed on the GaOOH surface, where the NO group dissociates upon adsorption.

**Figure S4.** In contrast to GaOOH, RSNO adsorption on Ga_2_O_3_ preserves the NO group, indicating lower catalytic activity**.** Optimized structure of Ph-SNO adsorbed on the Ga_2_O_3_ surface, showing intact NO group after adsorption.

**Figure S5.** Optical image of TA/Ga nanoparticle pellet.

**Figure S6.** TEM image of TA/Ga nanoparticles, with corresponding TEM-EDS elemental maps showing the distribution of C, Ga, and O atoms. Scale bar = 100 nm.

**Figure S7.** Optical image of Ta-Zr^4+^ gel assembly.

**Figure S8.** TA-Zr^4+^-Ga coatings form uniform and substrate-independent films across different materials. Optical images comparing uncoated and TA-Zr^4+^-Ga-coated samples of aluminum and PVC/DEHP substrates.

**Figure S9.** Schematic illustration of the coating process for a Ti wire, and SEM images of uncoated and TA-Zr⁴⁺-Ga-coated Ti wires. Scale bar = 50 μm and 5 μm.

**Figure S10.** Representative images of the contact angle measurements of TA-Zr^4+^-Ga-1 layer, TA-Zr^4+^-Ga-2 layer, and TA-Zr^4+^ coatings on bare glass substrates.

**Figure S11.** X-ray photoelectron spectroscopy (XPS) survey spectrum of the TA-Zr^4+^-Ga coating.

**Figure S12.** Fluorescence microscopy images of RAW 264.7 cells after 24 h of incubation on glass (CT), TA-Zr^4+^, TA-Zr^4+^-Ga_1_ (1 layer), and TA-Zr^4+^-Ga_2_ (2 layers). Cells stained with calcein (green) indicate live cells and ethidium (red) indicate dead cells. Scale bar = 100 μm.

**Figure S13.** TA-Zr^4+^-Ga coatings on glass substrate after 1 spin cycle (1 layer), 2 spin cycles (2 layers), and 3 spin cycles (3 layers).

**Figure S14.** Quantification of Ga loading per TA-Zr⁴⁺-Ga-coated glass substrate (1 layer) by ICP-OES. Data are presented as mean ± standard deviation from three independently prepared coated substrates (n = 3).

24h

48h

**Figure S15.** Cell viability of HUVECs after 24-48 h of incubation on glass (CT), TA-Zr^4+^, and TA-Zr^4+^-Ga1 (1 layer), as determined using the Alamar Blue assay. Data are presented as mean ± standard deviation from three independent experiments (n = 3).

**Figure S16.** Cell viability of HUVECs after 24 h of incubation on glass (CT), TA-Zr^4+^, and TA-Zr^4+^-Ga1 (1 layer), as determined using the CCK-8 assay. Data are presented as mean ± standard deviation from three independent experiments (n = 3).

**Figure S17.** Hemocompatibility of developed films evaluated by hemolysis assay following ISO 10993-4 guidelines. a. Hemolysis (%) was calculated relative to the positive control (water, 100% hemolysis), with HDPE used as the negative reference material. Data are presented as mean ± standard deviation, n = 3 independent donors, with 3 replicates per donor. All coatings exhibited hemolysis levels below 5%, indicating good hemocompatibility. b. Representative images of the samples incubated with red blood cells for 2 h at room temperature.

**Figure S18.** NO generation from TA-Zr^4+^ coatings with one, two, and three spin-coating cycles, measured using the Griess assay. Data are presented as mean ± standard deviation from three independently prepared coated samples (n = 3).

**Figure S19.** Quantification of NO generation from TA-Zr^4+^-Ga_1_, TA-Zr^4+^-Ga_2_, TA-Zr^4+^, and bare glass (CT) incubated with HUVEC culture medium containing GSNO (50 µM). Data are from one independent experiment and are presented descriptively; no inferential statistical analysis was performed.

**Figure S20**. Reusability of TA-Zr^4+^-Ga coating over five consecutive GSNO incubation cycles using the same coated sample, demonstrating sustained NO-generating activity. Data are presented as mean ± standard deviation (n = 3).

**Figure S21.** XPS spectra of TA-Zr^4+^-Ga coating before and after 5 GSNO incubation cycles, showing preservation of the characteristic Ga, Zr, O, and organic signals following repeated testing. Samples were gently rinsed with water prior to analysis.

**Figure S22.** Cumulative Ga release from TA-Zr^4+^-Ga-coated glass coverslips during incubation in PBS supplemented with serum for up to 10 days, as determined by ICP-MS. Supernatants were collected at defined time points, and cumulative release was calculated by accounting for both the remaining solution volume and previously collected aliquots. Data are presented as mean ± standard deviation from three independently prepared coated samples (n = 3).

**Figure S23.** SEM micrographs of TA-Zr^4+^-Ga coating before (a) and after incubation in PBS supplemented with serum for 1 day (b) and 7 days (c). The coating retained its overall morphology and surface coverage, with no evidence of significant degradation or delamination following prolonged incubation. Scale bar = 1 μm.

**Figure S24**. UV-vis spectra of TA-Zr^4+^-Ga coatings on quartz substrates after 1 h exposure to pH 3, pH 7.4 (PBS, control), pH 9, and sonication (5 min). The characteristic TA-Zr coordination band (~350 nm) and tannic acid absorption features are retained following treatment, indicating preservation of coating components.

**Figure S25.** Confocal microscopy images showing localized intracellular NO signals in HUVECs cultured on TA-Zr^4+^-Ga_1_ and TA-Zr^4+^-Ga_2_, visualized using the NO-sensitive fluorescent probe DAF-FM. Scale bar = 50 μm.

**Figure S26.** Microscopy images showing localized areas of NO release signals in HUVECs cultured on TA-Zr^4+^-Ga_2_. Scale bar = 50 μm.

**Figure S27**. Representative fluorescence images of intracellular ROS levels in RAW 264.7 macrophages assessed by DCFH-DA staining under LPS stimulation. Control cells (CT) show low basal ROS levels, while LPS treatment (CT + LPS) induces a significant increase in ROS generation. Cells treated with TA-Zr^4+^ and TA-Zr^4+^-Ga coatings in the presence of LPS exhibit a marked reduction in fluorescence intensity, indicating effective suppression of intracellular oxidative stress. Scale bar = 200 µm.

**Figure S28.** Representative confocal microscopy images of RAW 264.7 macrophages under LPS stimulation following treatment with TA-Zr⁴⁺ and TA-Zr⁴⁺-Ga coatings. Immunofluorescence staining shows NF-κB (green) and IL-10 (red), with nuclei counterstained using Hoechst (blue). Scale bar = 50 µm.

**Figure S29.** Quantification of relative total cellular NF-κB p65 fluorescence intensity in RAW 264.7 macrophages under LPS stimulation following treatment with TA-Zr^4+^ and TA-Zr^4+-^Ga coatings. Statistical significance was assessed using one-way ANOVA followed by Tukey’s multiple-comparisons test. **p* < 0.05, ***p* < 0.01, ****p* < 0.001, and *****p* < 0.0001. Data are presented as mean ± standard deviation from three independent biological experiments (n = 3).

**Figure S30.** Quantification of IL-10 expression in RAW 264.7 macrophages under LPS stimulation following treatment with TA-Zr^4+^ and TA-Zr^4+^-Ga coatings. Statistical significance was assessed using one-way ANOVA followed by Tukey’s multiple-comparisons test. ns = non-significant, **p* < 0.05, ***p* < 0.01, ****p* < 0.001, and *****p* < 0.0001. Data are presented as mean ± standard deviation from three independent biological experiments (n = 3).
